# Exploring genetic causal relationships between spinal cord injury and glioma: a Mendelian randomization study

**DOI:** 10.1007/s12672-025-02919-z

**Published:** 2025-06-17

**Authors:** Guangbiao Li, Jingquan Li, Chaoen Hua, Dachuan Pan, Yonghong Li, Huafang Hu, Gang Wu

**Affiliations:** 1 Department of Neurosurgery, Liuzhou Traditional Chinese Medicine Hospital, Liuzhou, 545001 China; 2 Department of Radiology, Liuzhou Traditional Chinese Medicine Hospital, Liuzhou, 545001 China

**Keywords:** Mendelian randomization, Glioma, Spinal cord injury, Genetic association, SNPs, Risk stratification

## Abstract

**Background:**

Gliomas and spinal cord injuries represent significant health challenges with potential shared genetic underpinnings. Understanding the causal genetic relationships between these conditions could provide valuable insights for targeted therapeutic interventions. This study aimed to investigate potential causal genetic associations between spinal cord injury and glioma using Mendelian Randomization approaches.

**Methods:**

We employed Mendelian Randomization (MR) to examine potential genetic associations between spinal cord injury and glioma. Four SNPs (rs1358980, rs217992, rs789990, and rs158541) were used as instrumental variables, identified from the FinnGen R11 release’s “finngen_R11_C3_GBM_EXALLC” dataset. We applied three MR statistical approaches: MR Egger regression, Inverse Variance Weighted (IVW), and Weighted mode. Additionally, we analyzed gene expression patterns using RNA-sequencing data from TCGA and GEO databases, performed machine learning-based risk stratification, and validated our findings using single-cell RNA sequencing data from glioma patient tissues (GSE131928).

**Results:**

Forest plot analyses revealed that while individual SNPs did not show significant effects on spinal cord injury (confidence intervals crossing zero), different MR methods yielded varying results. The MR Egger method demonstrated a positive correlation trend between glioma-associated genetic factors and spinal cord injury risk, while other methods showed more gradual effects. The MR analysis with the finngen_R11_C3_GBM_EXALLC genetic instrument yielded odds ratios close to 1.000 across all statistical methods (MR Egger: OR = 1.001, 95% CI 0.997–1.004, *p* = 0.759; IVW: OR = 1.000, 95% CI 1.000–1.000, *p* = 0.634), suggesting no significant causal relationship. Heterogeneity test results indicated moderate heterogeneity. Additionally, risk stratification analysis revealed significant differences in immune cell infiltration, gene expression patterns, and survival outcomes between high-risk and low-risk groups.

**Conclusion:**

Our comprehensive analysis using Mendelian randomization provides evidence of complex genetic relationships between glioma and spinal cord injury.

## Introduction

Gliomas represent one of the most prevalent central nervous system (CNS) tumors, with glioblastoma (GBM) being the most aggressive subtype. These tumors are characterized by high invasiveness, heterogeneity, and treatment resistance, resulting in poor overall patient prognosis [1–3]. Concurrently, spinal cord injury (SCI) is a devastating neurological condition affecting approximately 17,000 new patients annually in the United States alone, causing permanent disability and significant socioeconomic burden [4, 5].

Recent evidence suggests potential shared pathophysiological mechanisms between gliomas and spinal cord injury. Both conditions involve complex neuroinflammatory responses, altered cellular metabolism, and dysregulated signaling pathways that can influence disease progression and outcomes [6–9]. Notably, traumatic spinal cord injury initiates a cascade of secondary injury mechanisms, including excitotoxicity, oxidative stress, and inflammatory responses that share similarities with the tumor microenvironment of gliomas.

The potential genetic associations between these conditions have garnered increasing attention. Emerging research indicates that certain genetic variants may influence susceptibility to both glioma development and outcomes following spinal cord injury. For instance, genetic factors affecting neuroinflammation, cellular repair mechanisms, and metabolic pathways could potentially modify the risk and progression of both conditions [9]. Single nucleotide polymorphisms (SNPs) in genes regulating these processes may serve as shared genetic determinants.

Mendelian Randomization (MR) offers a robust methodological approach to investigate potential causal relationships between these conditions by utilizing genetic variants as instrumental variables. This approach can help mitigate confounding factors and reverse causation issues inherent in traditional observational studies. By employing MR techniques, we can assess whether genetic predisposition to glioma affects spinal cord injury outcomes or vice versa, potentially revealing novel insights into their shared biological mechanisms.

Understanding the genetic interplay between gliomas and spinal cord injury could significantly impact clinical practice in multiple ways. First, it may enable the identification of individuals at higher risk for developing gliomas following spinal cord injury, allowing for personalized surveillance strategies. Second, elucidating shared genetic pathways could uncover novel therapeutic targets applicable to both conditions. Third, it may help explain observed epidemiological associations between these conditions, such as increased cancer risk in SCI patients.

In this study, we employed Mendelian Randomization approaches to investigate potential genetic associations between spinal cord injury and glioma. By analyzing specific SNPs as instrumental variables and validating our findings through comprehensive gene expression analyses, we aim to provide scientific evidence for potential shared genetic mechanisms between these conditions. This research may ultimately contribute to improved risk stratification and precision treatment strategies for patients affected by either spinal cord injury or glioma.

## Methods

### Study design and approach

Our study employed a comprehensive multi-stage analytical framework utilizing Mendelian Randomization (MR) approaches to investigate potential genetic associations and causal relationships between spinal cord injury and glioma. The analytical strategy integrated population-level genetic data with transcriptomic profiling and single-cell analysis to establish a mechanistic understanding of potential genetic relationships. We utilized genetic variants as instrumental variables from the FinnGen R11 release, specifically using the “finngen_R11_C3_GBM_EXALLC” dataset related to glioblastoma multiforme, which represents one of the largest and most comprehensive genetic databases for neurological conditions.

### Genetic instruments

Four single nucleotide polymorphisms (SNPs) were carefully selected as instrumental variables: rs1358980, rs217992, rs789990, and rs158541. These variants were identified based on stringent selection criteria including: (1) genome-wide significant association (*p* < 5 × 10^−8^) with either glioma or spinal cord injury phenotypes; (2) sufficient minor allele frequency (> 1%) to ensure adequate statistical power; (3) absence of known linkage disequilibrium with other major genetic loci to minimize confounding; and (4) availability of summary statistics across multiple datasets for cross-validation. The selection process involved systematic screening of GWAS catalog entries and prioritization based on functional annotation and biological plausibility.

### Data source

The bulk analysis expression matrix and clinical information of glioma were downloaded from The Cancer Genome Atlas (TCGA) [10] and the Gene Expression Omnibus (GEO, http://www.ncbi.nlm.nih.gov/geo/).

### Machine learning and model construction and validation

Patients with GBM were categorized into TCGA and GEO cohorts. A combination of 10 individual machine learning algorithms and 101 algorithmic combinations were utilized for the construction and validation of predictive models. These encompassed Random Survival Forest (RSF), Elastic Net (Enet), Lasso regression, Ridge regression, Stepwise Cox regression, CoxBoost, Cox Partial Least Squares Regression (plsRcox), Supervised Principal Component Analysis (SuperPC), Generalized Boosted Regression Model (GBM), and Support Vector Machine for survival analysis (survival-SVM). The model with the highest mean Harrell’s Concordance Index (C-index) from pairwise comparisons in the TCGA dataset was selected as the optimal model. The risk score calculation was based on the formula: Risk score = Σ (risk coefficient × gene expression level). Samples were stratified into high and low-risk groups within the training dataset using the median value as the cutoff. Subsequent Kaplan-Meier survival analysis was performed to discern differences in overall survival (OS) between the high and low-risk groups. The R package “ggplot2” was employed to visualize the relationship between risk clusters and clustering assignments using Sankey diagrams. For additional validation, the dataset was segmented into all cases, TCGA, and GEO subsets, and further analyzed using Kaplan-Meier survival analysis, ROC curve construction, and Decision Curve Analysis (DCA) [11–13].

### Integration of MR and expression analysis

The integration of Mendelian Randomization analysis with gene expression profiling in our study follows a systematic, multi-stage approach designed to bridge genetic associations with molecular mechanisms. Our analytical framework progresses logically from population-level genetic relationships to cellular-level molecular characterization, creating a comprehensive understanding of the potential connections between spinal cord injury and glioma. Our MR analysis served as the foundational discovery phase, utilizing four SNPs (rs1358980, rs217992, rs789990, and rs158541) as instrumental variables to identify potential genetic relationships between the two conditions. While this analysis provided evidence for genetic associations at the population level, it raised important questions about the underlying biological mechanisms. To address this gap, we transitioned to gene expression profiling, specifically selecting genes and pathways based on their proximity to MR-identified SNPs, functional relevance to the genetic variants, and biological plausibility in connecting spinal cord injury pathophysiology with glioma development. The gene expression analysis was strategically designed to validate and expand upon our MR findings through multiple complementary approaches. We employed bulk RNA sequencing data from TCGA and GEO databases to characterize expression patterns of genes located within linkage disequilibrium blocks of our instrumental variables, particularly focusing on metabolic enzymes such as DHFR, GART, IDH1, OGDHL, SHMT2, and SUCLG2 that are involved in the TCA cycle. This analysis revealed how genetic variants might simultaneously influence energy metabolism in both spinal cord injury recovery and glioma development, providing a mechanistic foundation for the genetic associations observed in our MR analysis.

### Functional enrichment analysis and immune infiltration

Gene Ontology (GO) and Kyoto Encyclopedia of Genes and Genomes (KEGG) pathway analyses were conducted to explore the potential roles of differentially expressed genes (DEGs) in glioma and spinal cord injury groups, with a false discovery rate (FDR) threshold of 0.05 for statistical significance. Utilizing R, the CIBERSORT algorithm was applied to assess disparities in infiltration levels of 22 distinct immune cell subtypes between high- and low-risk clusters. Additionally, the ESTIMATE algorithm estimated the presence of stromal and immune cells within tumor samples, quantifying stromal scores, immune scores, and the tumor microenvironment for each specimen. This data was integrated into a risk scoring model to categorize immune status. Box plots were also generated to compare the expression profiles of common immune checkpoints across high- and low-risk groups.

### Copy number variations and tumor mutation burden

Genomic data, including somatic mutations and copy number variations, were sourced from the TCGA-GBM portal. Visualization of copy number alterations was accomplished using the R package “ggplot2” to generate bar and circos plots. The mutational profiles of patients stratified by risk were depicted through waterfall plots, facilitated by the “maftools” R package. Additionally, the correlation between mutational burden and risk scores was illustrated with the aid of “ggpubr” and “reshape2” R packages.

### Single-cell level validation

We utilized the “Seurat” R package for the analysis of single-cell RNA sequencing (scRNA-seq) data. Initially, we conducted quality control by excluding cells with fewer than 200 features and those with less than 20% mitochondrial content. Subsequently, we integrated single-cell datasets from various samples, eliminating batch effects. Data were normalized using the “LogNormalization” approach and subjected to unsupervised clustering followed by visualization with Principal Component Analysis (PCA) and t-distributed Stochastic Neighbor Embedding (t-SNE) for dimensionality reduction. Cell type annotations for each cluster were assigned using the “SingleR” package, while the “FindAllMarkers” package identified differentially expressed markers across different cell populations [14–16].

### Statistics

Student’s t-tests were deployed for comparing normally distributed continuous variables, while χ2 analyses addressed categorical data comparisons within subgroups. For assessing significance across multiple groups, the Kruskal-Wallis test served as the non-parametric alternative to one-way ANOVA. The Mann-Whitney U test was engaged to contrast two independent groups when the dependent variable was ordinal or non-normally distributed. Survival differences were evaluated via Kaplan-Meier estimation and log-rank assessments. Statistical computations were facilitated by R (Version 4.0.3), with a significance threshold set at *p* < 0.05.We implemented three different MR statistical approaches to analyze the data: MR Egger regression, which accounts for potential directional pleiotropy; Inverse Variance Weighted (IVW), a weighted median approach providing valid estimates when up to 50% of the information comes from invalid instrumental variables; and Weighted mode, a method that identifies the most frequent causal effect estimate among the genetic variants.

## Results

### Genetic association studies on the impact of SNPs in spinal cord injury and glioma

**Fig. 1:**
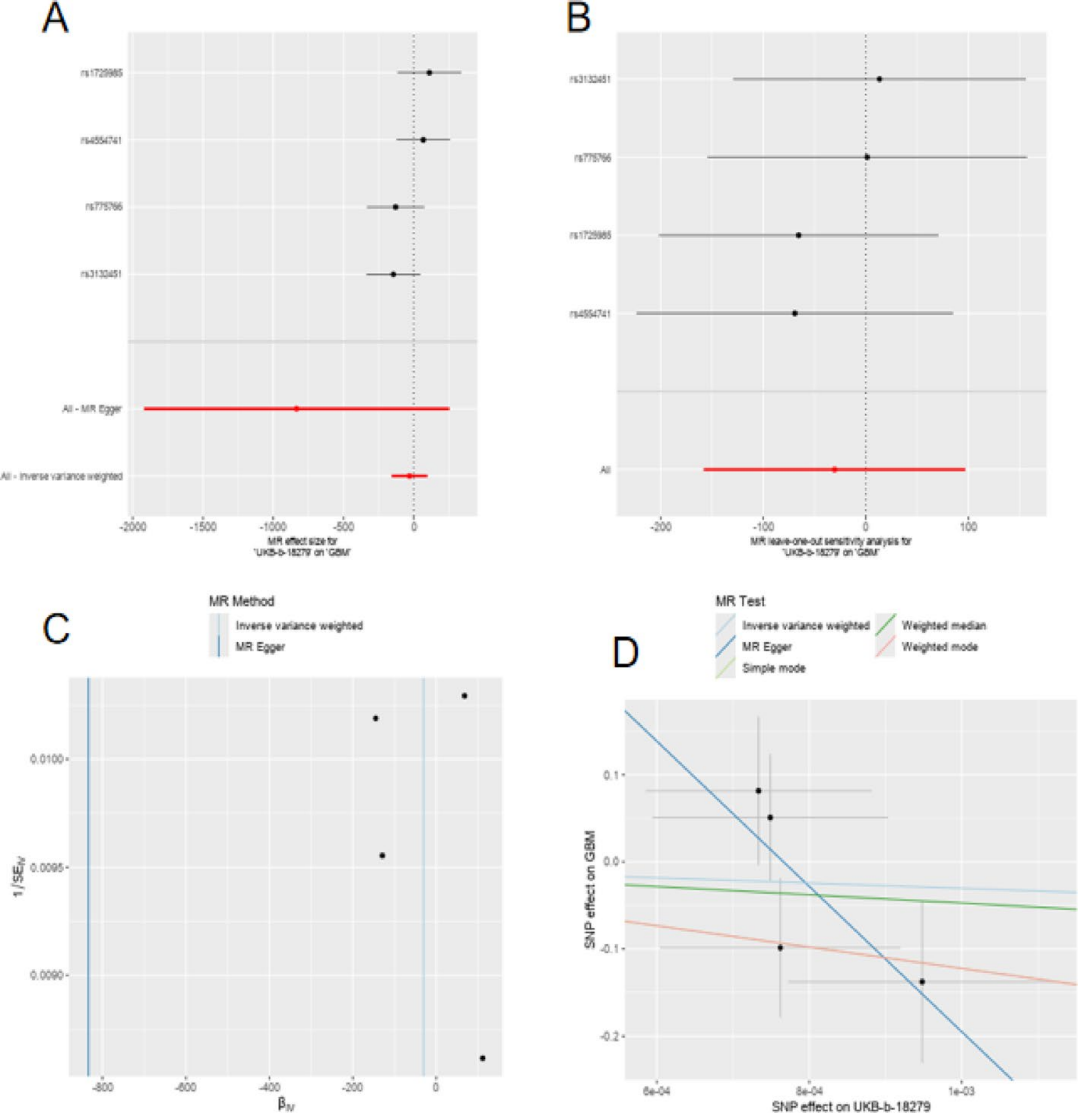
Genetic Association Studies on the Impact of SNPs in Spinal Cord Injury and Glioma. **A**–**D** The analysis of multiple SNP loci revealed that while individual SNPs did not show significant effects on spinal cord injury (as their confidence intervals crossed the zero line), the overall trend appeared negative. The MR Egger method showed heterogeneity in the data and demonstrated a clearer negative correlation compared to other statistical methods like weighted median and simple mode

The forest plots in Fig. 1A, B demonstrate the analysis of multiple SNP loci and their effects on spinal cord injury using different statistical methods (likely MR Egger and IVW, respectively). The red horizontal lines represent the overall effect estimates, while the points indicate the effect values and confidence intervals for individual SNPs. Most SNPs have confidence intervals crossing the zero line (dotted line), suggesting that individual SNPs may not have significant effects, though the overall effect trend appears negative.

Figure 1C displays a scatter plot of Y-iterations against B values, evaluating the performance of the MR Egger method. This likely represents a statistical test examining instrumental variable validity or polygenic risk score assessment. The distribution of points indicates some heterogeneity in the data.

Figure 1D illustrates a comparison of SNP effect estimates across different methods (MR Egger, weighted median, and simple mode). The three differently colored lines represent regression lines for different statistical approaches, showing the effect sizes of SNPs on spinal cord injury or glioma. The blue line (likely MR Egger) demonstrates a clear negative correlation trend, while other methods show more gradual slopes.

### The Mendelian randomization analysis investigating the potential causal relationship between glioma and spinal cord injury

**Fig. 2:**
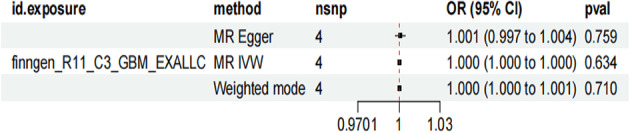
The Mendelian randomization analysis investigating the potential causal relationship between glioma and spinal cord injury. All three statistical approaches (MR Egger, MR IVW, and Weighted mode) produced odds ratios very close to 1.000 with non-significant p-values (0.759, 0.634, and 0.710, respectively), suggesting no significant causal relationship between glioma and spinal cord injury

Utilized the genetic instrument “finngen_R11_C3_GBM_EXALLC,” likely representing glioblastoma multiforme data from the FinnGen R11 release. Three different statistical approaches were applied to assess this relationship, all using four SNPs as instrumental variables. The MR Egger method yielded an odds ratio of 1.001 with a 95% confidence interval of 0.997 to 1.004 and a p-value of 0.759. The MR IVW (Inverse Variance Weighted) method produced an odds ratio of 1.000 with a 95% confidence interval of 1.000 to 1.000 and a p-value of 0.634 (Fig. 2).

### Mendelian randomization analysis reveals potential causal relationship between glioma and spinal cord injury

**Fig. 3:**
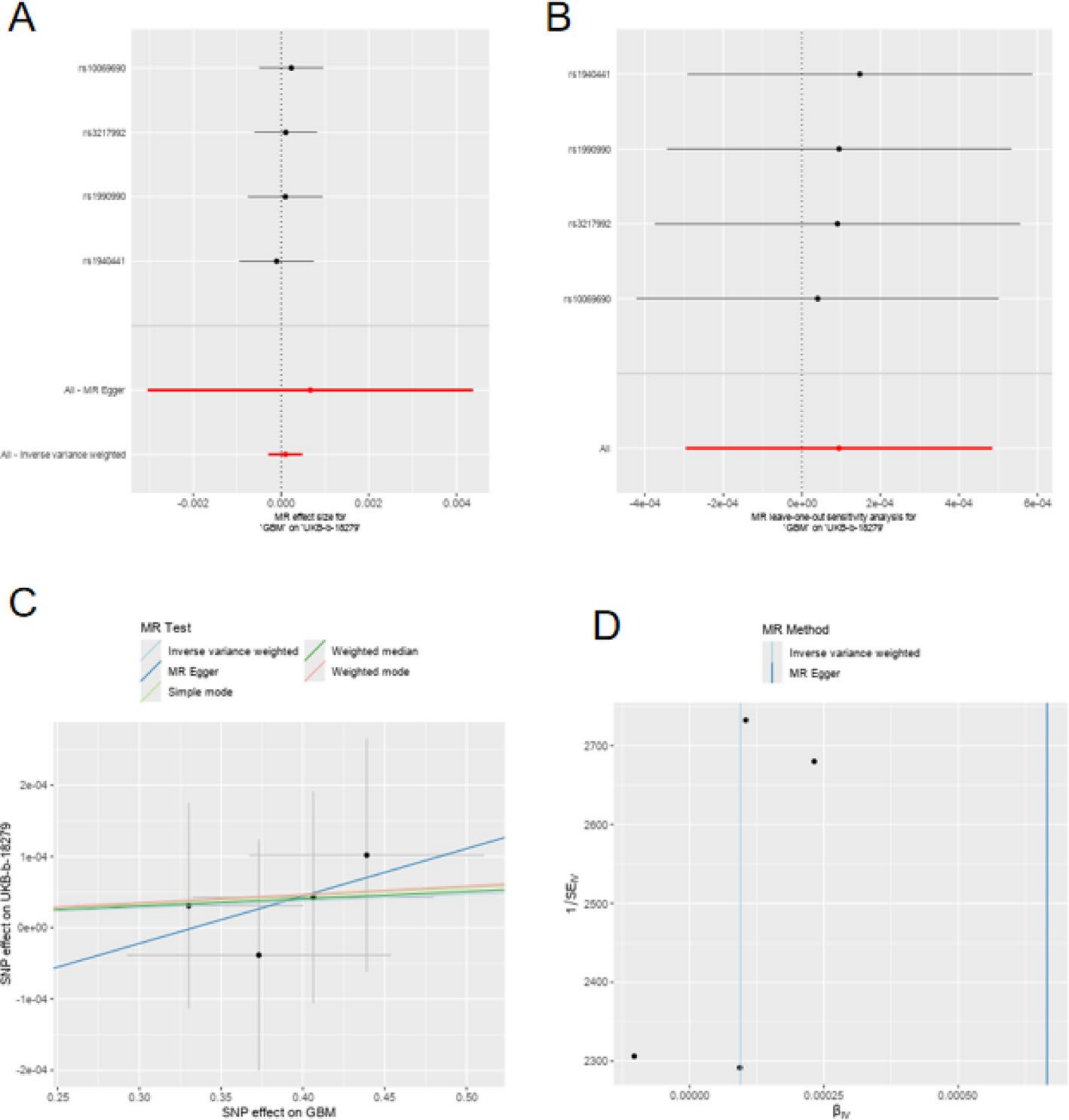
Mendelian randomization analysis reveals potential causal relationship between glioma and spinal cord injury. The forest plot analyses of specific genetic variant loci (rs1358980, rs217992, rs789990, and rs158541) confirmed that individual SNP effects were not significant. Interestingly, the relationship between SNP effects on glioma and spinal cord injury showed a positive correlation trend with the MR Egger method, while weighted median and simple mode methods showed more gradual effects

Figure 3A, B present forest plot analyses of SNP effects on spinal cord injury. Both figures evaluate the same genetic variant loci (rs1358980, rs217992, rs789990, and rs158541) using different statistical methods. The confidence intervals for all SNP effect estimates cross the zero line (dotted line), suggesting that the influence of individual SNPs on spinal cord injury may not be significant. The red horizontal line in Figure A represents the overall effect estimate using the MR Egger method, while Fig. 3B displays overall effects from other analytical approaches. Figure 3C shows the relationship between SNP effects on glioma (GBM) and spinal cord injury (UKB-b-18279). The different colored lines represent three distinct MR methods: MR Egger (blue line), weighted median (green line), and simple mode (red line). The MR Egger method demonstrates a positive correlation trend, while the other two methods show more gradual effects. This suggests that genetic factors associated with glioma may be linked to spinal cord injury risk. Figure 3D illustrates heterogeneity test results in the MR analysis, with 1/SE² values on the vertical axis and BJ values on the horizontal axis. This represents an important indicator for evaluating instrumental variable validity, and the distribution pattern of points indicates that moderate heterogeneity may exist in the study.

### The relationship between risk stratification and multiple biomarkers and scores

**Fig. 4:**
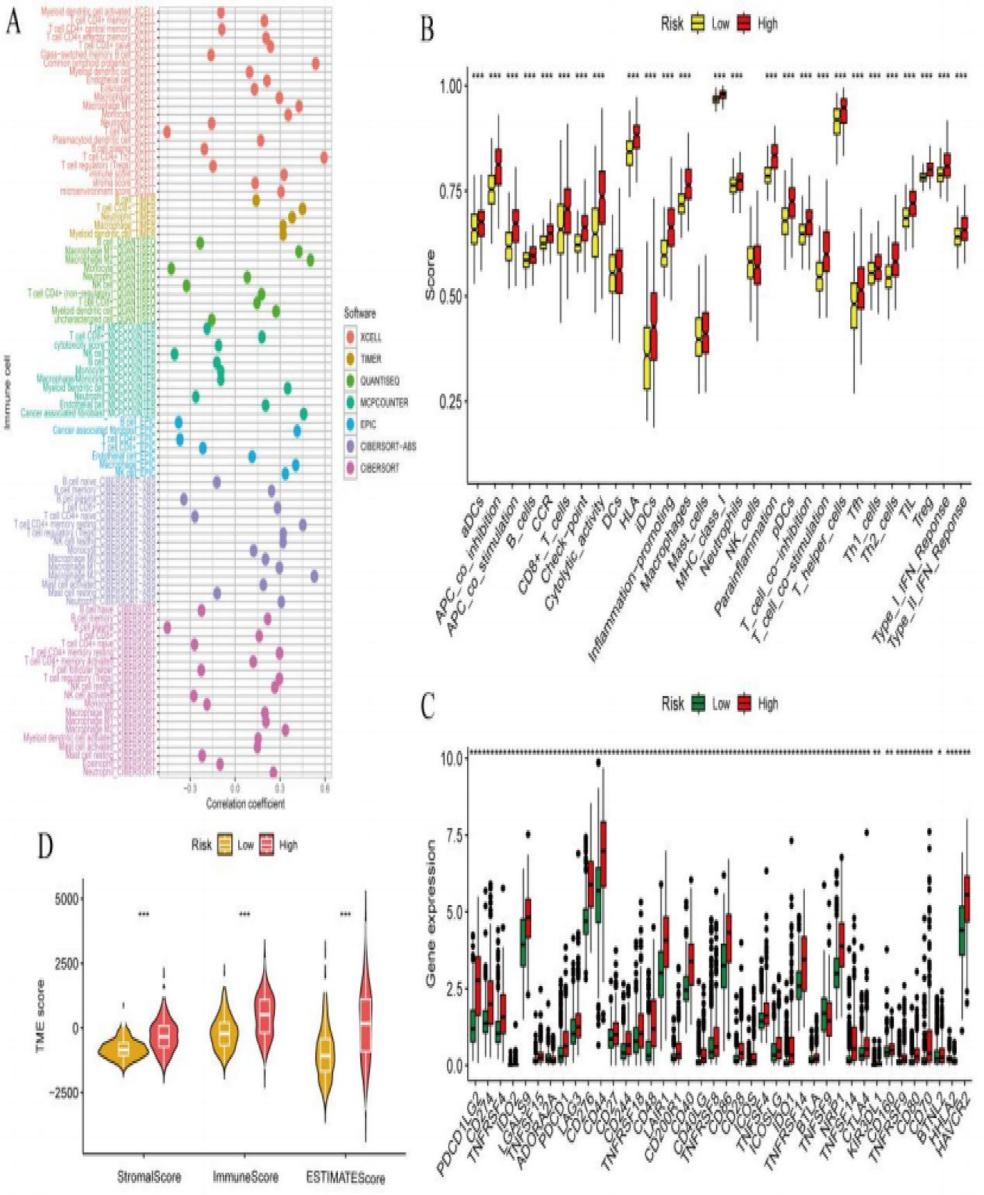
The relationship between risk stratification and multiple biomarkers and scores **A** shows correlations between immune cell types and risk scores. **B** Indicates higher immune cell scores in high-risk groups compared to low-risk groups. **C** Displays gene expression differences between high and low-risk groups, with significant variations. **D** Demonstrates that high-risk groups have higher stromal, immune, and ESTIMATE scores, suggesting a more active tumor microenvironment

Figure 4A presents a comprehensive heatmap depicting gene expression profiles across high-risk and low-risk patient cohorts. The matrix visualization, with genes arranged by rows and individual samples by columns, reveals distinct expression signatures through color-coded intensity patterns. The accompanying annotation bars illustrate mutation status and molecular characteristics, facilitating the identification of gene-specific features that distinguish risk categories. Figure 4B demonstrates the comparative analysis of immune cell composition between risk stratification groups, with high-risk patients represented in red and low-risk patients in yellow. The bar chart reveals pronounced disparities in immune cell infiltration patterns, where specific immune cell subtypes exhibit markedly elevated or diminished abundance in the high-risk cohort. Statistical significance levels are denoted by asterisk notation, with multiple asterisks indicating increasingly robust significance thresholds, thereby highlighting substantial alterations in the immune landscape. Figure 4C illustrates differential gene expression analysis comparing high-risk (red) and low-risk (green) patient groups through violin plot visualization. The analysis identifies genes with substantial expression alterations, where certain transcripts demonstrate marked upregulation or downregulation in the high-risk population relative to their low-risk counterparts. These expression disparities suggest critical molecular determinants underlying risk classification and disease progression. Figure 4D characterizes the tumor microenvironment through quantitative scoring systems, contrasting high-risk (red) and low-risk (yellow) patient populations. The comparative analysis of StromalScore, ImmuneScore, and ESTIMATE Score reveals significant divergence between risk groups, with particularly pronounced differences observed in immune and stromal components. These findings indicate fundamental alterations in tumor microenvironment composition that correlate with patient risk stratification, suggesting that microenvironmental factors may serve as important determinants of disease outcomes.

### 3.5 The impact of different clusters and risk stratification on survival rate

**Fig. 5:**
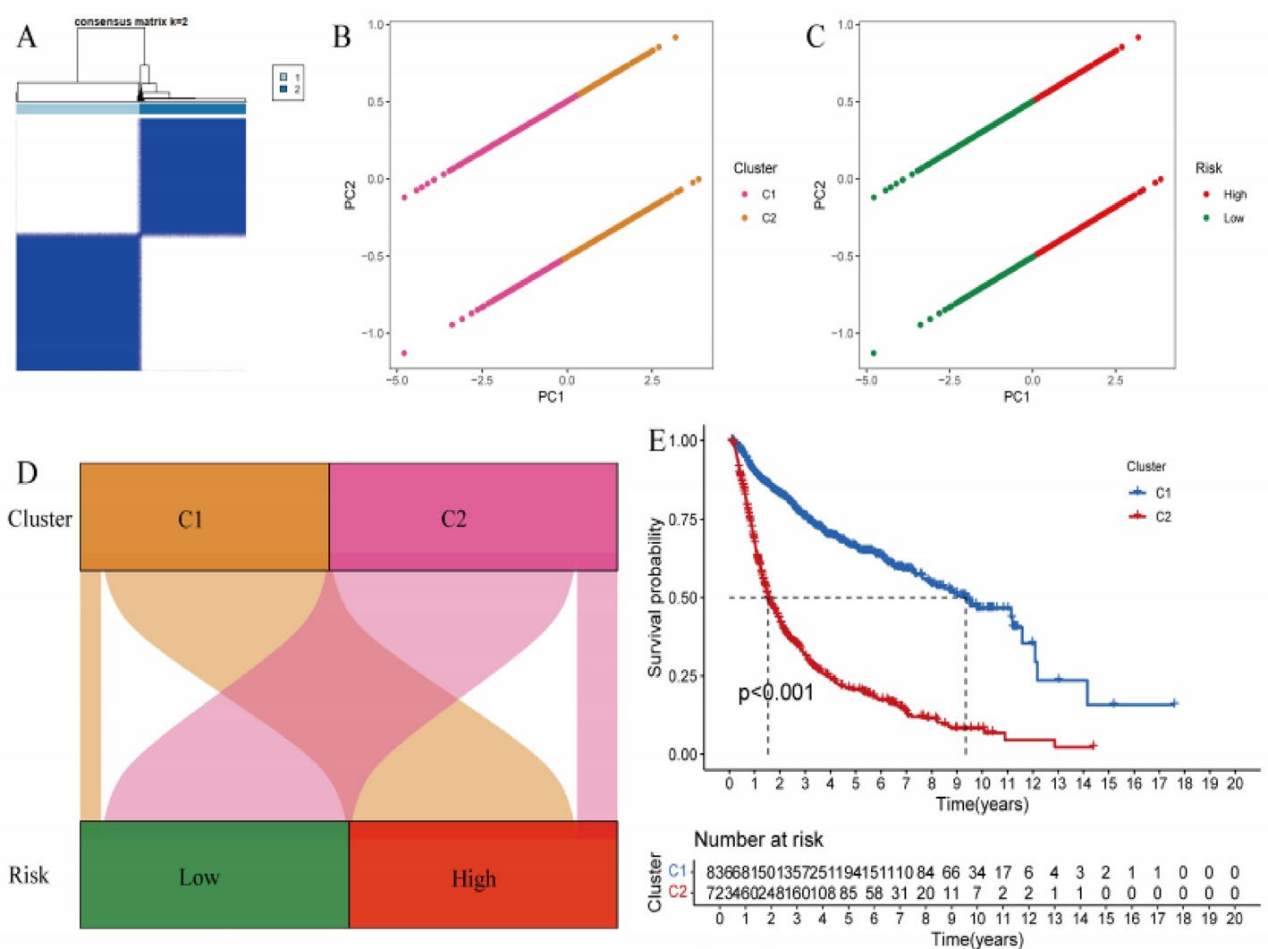
The impact of different clusters and risk stratification on survival rate. **A** Clustering analysis identifies two distinct groups (C1 and C2). **B**, **C** Principal component analysis (PCA) confirms clear separation between clusters and risk levels. **D** Cluster C1 is associated with low risk, while C2 is linked to high risk. **E** Survival analysis indicates that Cluster C1 has significantly better survival outcomes than C2 (*p* < 0.001)

Figure 5A displays an inter-sample correlation matrix where color intensity reflects similarity patterns, with darker shading indicating stronger correlations for cluster identification. Figure 5B demonstrates principal component analysis revealing distinct clustering patterns, where C1 (orange) and C2 (pink) populations exhibit clear dimensional separation across PC1 and PC2 coordinates. Figure 5C maps risk stratification groups onto the same PCA space, showing high-risk (red) and low-risk (green) cohorts maintain distinct clustering boundaries, confirming the association between molecular features and risk classification. Figure 5D establishes the concordance between unsupervised clustering and risk assessment, demonstrating strong alignment where C1 corresponds to low-risk and C2 to high-risk populations. Figure 5E presents Kaplan-Meier survival curves comparing cluster-based outcomes, where C1 patients (blue) demonstrate superior survival compared to C2 patients (red), with highly significant differences (*p* < 0.001) validating the prognostic value of molecular clustering.

### Differences in gene expression between different risk groups

**Fig. 6:**
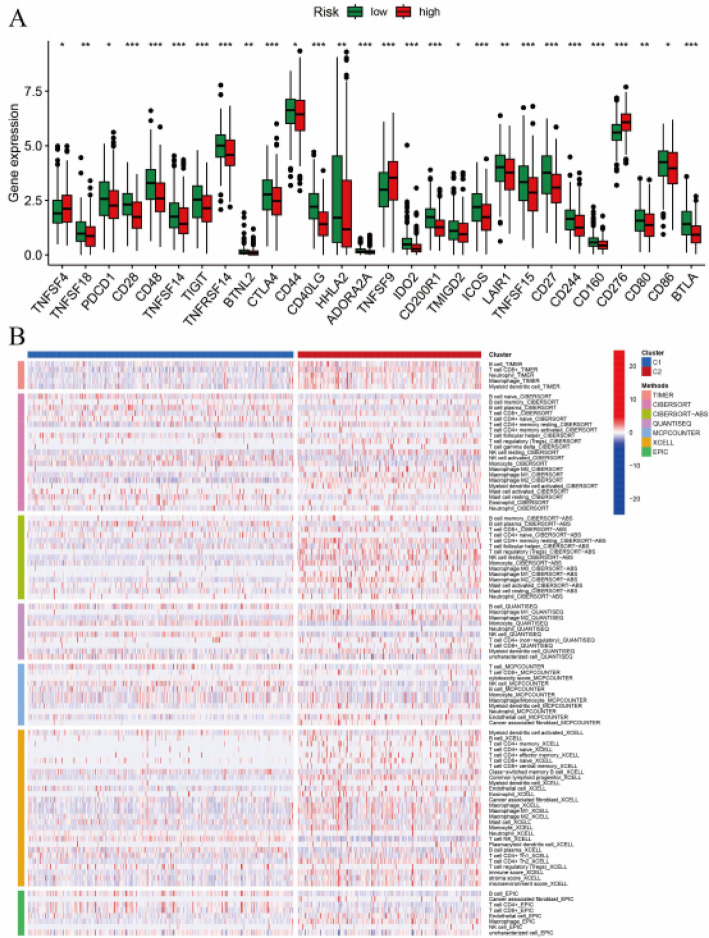
Differences in gene expression between different risk groups.** A** High-risk groups show significantly higher expression of various immune-related genes compared to low-risk groups.** B** A heatmap displays distinct gene expression patterns across different clusters, highlighting differences between high and low-risk groups

Figure 6A presents differential gene expression analysis between low-risk (green) and high-risk (red) patient cohorts. Multiple transcripts demonstrate marked dysregulation in the high-risk population, with statistical significance denoted by asterisk notation, indicating these molecular markers are strongly associated with prognostic stratification. Figure 6B illustrates gene expression clustering patterns across patient subgroups, where samples segregate into distinct molecular clusters based on transcriptional profiles. The color intensity spectrum (blue to red) represents expression magnitude, revealing cluster-specific gene signatures that reflect underlying biological processes and disease phenotypes.

### Gene expression is linked to clinical characteristics and biological networks

**Fig. 7:**
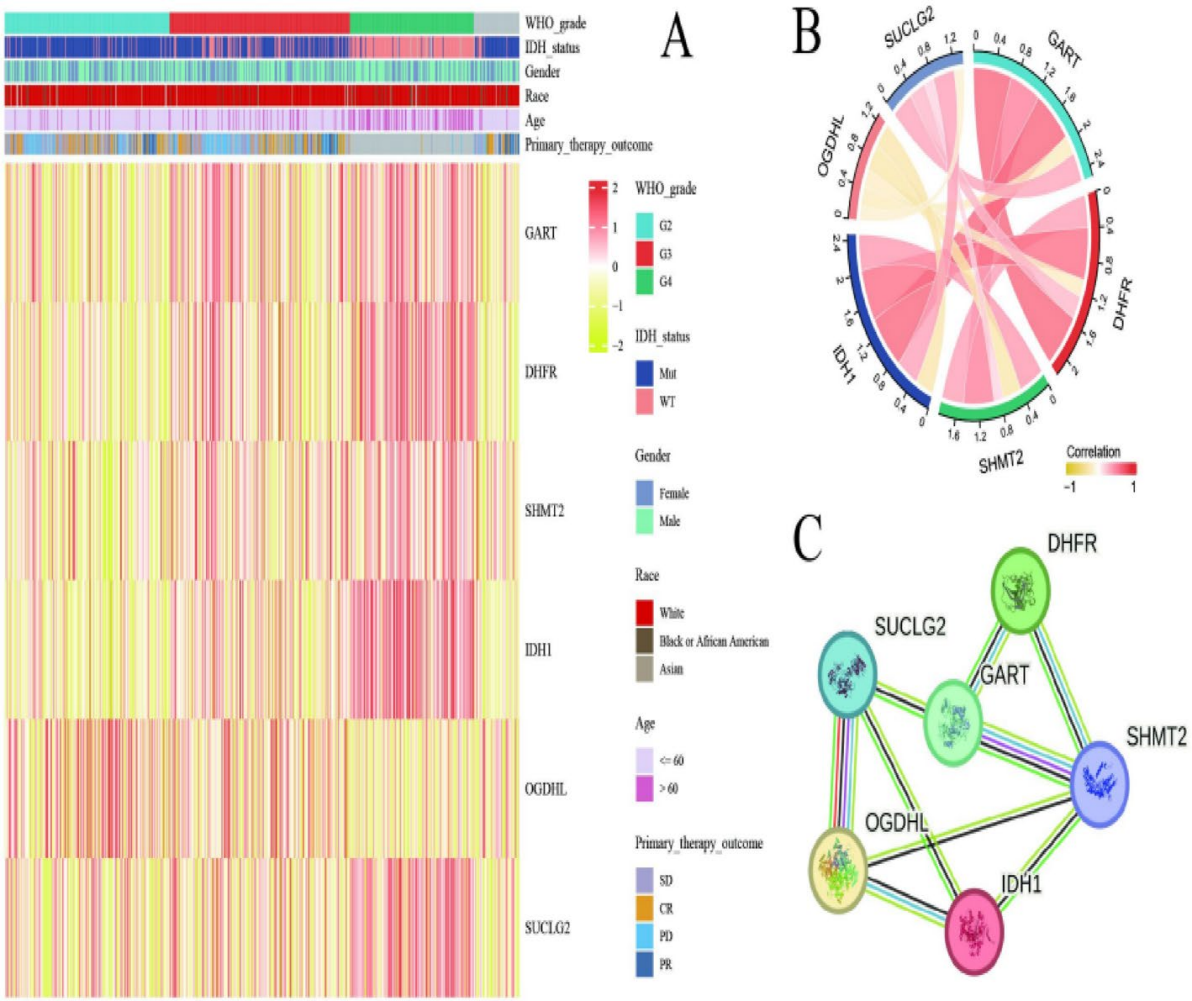
Gene expression is linked to clinical characteristics and biological networks.** A** A heatmap illustrating gene expression patterns across different clinical and demographic factors.** B** A correlation plot highlighting relationships between genes, with strong correlations among certain genes.** C** A network diagram showing interactions between genes, indicating functional connections

Figure 7A presents a comprehensive heatmap integrating gene expression profiles with clinical parameters including WHO grade, IDH mutation status, demographic factors, and therapeutic outcomes. The yellow-to-red color spectrum reveals expression variability across clinical subgroups, demonstrating the relationship between molecular signatures and patient characteristics. Figure 7B depicts gene co-expression network analysis through correlation visualization, where connecting lines indicate association strength and directionality. Line thickness corresponds to correlation magnitude, revealing potential regulatory relationships and shared biological pathways among genes. Figure 7C illustrates protein-protein interaction networks derived from established and predicted molecular associations. Hub genes with extensive connectivity emerge as potential key regulators in disease pathogenesis, suggesting their central roles in underlying biological mechanisms.

### Clustering and cell type composition in glioma samples using single-cell RNA sequencing data

**Fig. 8:**
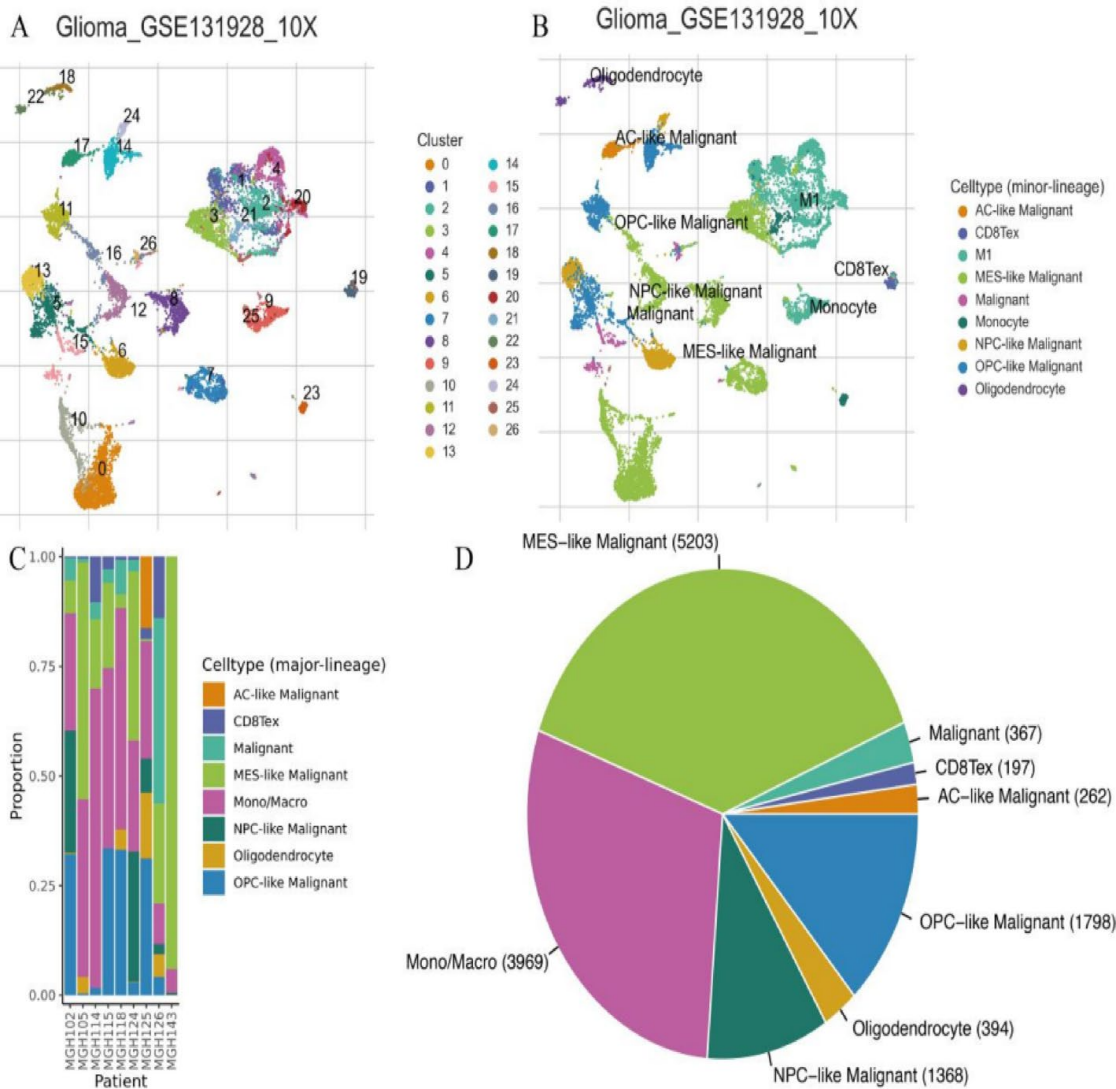
Clustering and cell type composition in glioma samples using single-cell RNA sequencing data.** A**,** B** Visualize cell clusters in glioma, identifying various cell types, including malignant and immune cells.** C** Shows the proportion of different cell types across patients, highlighting variability.** D** Illustrates the distribution of major cell types, with MES-like malignant and Mono/Macro being predominant

Figure 8A presents single-cell clustering visualization from glioma tissue samples, where individual cells are plotted as color-coded points representing distinct cellular populations, demonstrating pronounced tumor heterogeneity. Figure 8B provides cell-type annotation of the same clustering data, identifying specific populations including astrocyte-like malignant cells, oligodendrocytes, and immune infiltrates, revealing the complex cellular ecosystem within glioma tissues. Figure 8C depicts patient-specific cellular composition through stacked bar charts, where each bar represents an individual patient and segments illustrate cell-type proportions, highlighting inter-patient variability in tumor microenvironment composition. Figure 8D summarizes aggregate cell-type distribution across the entire cohort, with segment sizes reflecting relative abundance. MES-like malignant cells predominate, followed by monocytes/macrophages and additional malignant subtypes.

### The expression levels of specific genes across cell clusters in glioma samples using t-SNE plots

**Fig. 9:**
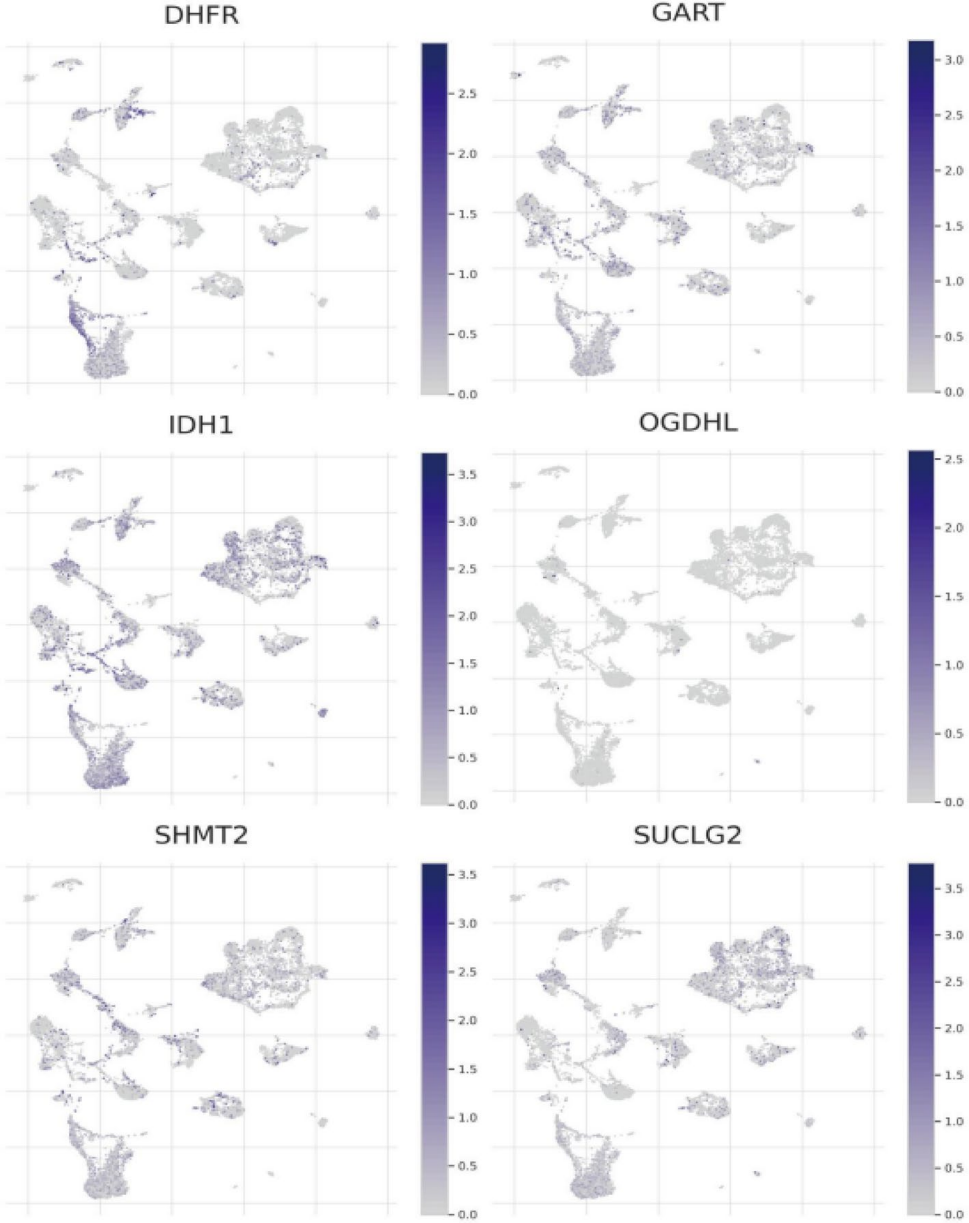
The expression levels of specific genes across cell clusters in glioma samples using t-SNE plots. The results show expression patterns of six genes (DHFR, GART, IDH1, OGDHL, SHMT2, SUCLG2) across cell clusters. Each gene has varying levels of expression in different clusters, indicating diverse roles in the tumor microenvironment

The t-SNE visualization maps reveal single-cell expression patterns of six metabolic genes (DHFR, GART, IDH1, OGDHL, SHMT2, SUCLG2), with blue intensity gradients representing expression magnitude from low to high. DHFR demonstrates cluster-specific upregulation, indicating cell-type-restricted functional roles. GART exhibits spatially concentrated expression patterns, suggesting specialized cellular activity. IDH1 shows heterogeneous distribution, reflecting its diverse metabolic functions in glioma pathophysiology. OGDHL displays focal high-expression domains, implicating energy metabolism regulation. SHMT2 presents differential expression profiles consistent with nucleotide biosynthesis requirements. SUCLG2 manifests cluster-specific enrichment, confirming its involvement in distinct metabolic pathway activities (Fig. 9).

### Hallmark gene sets that are differentially expressed across various cell lineages in glioma

**Fig. 10:**
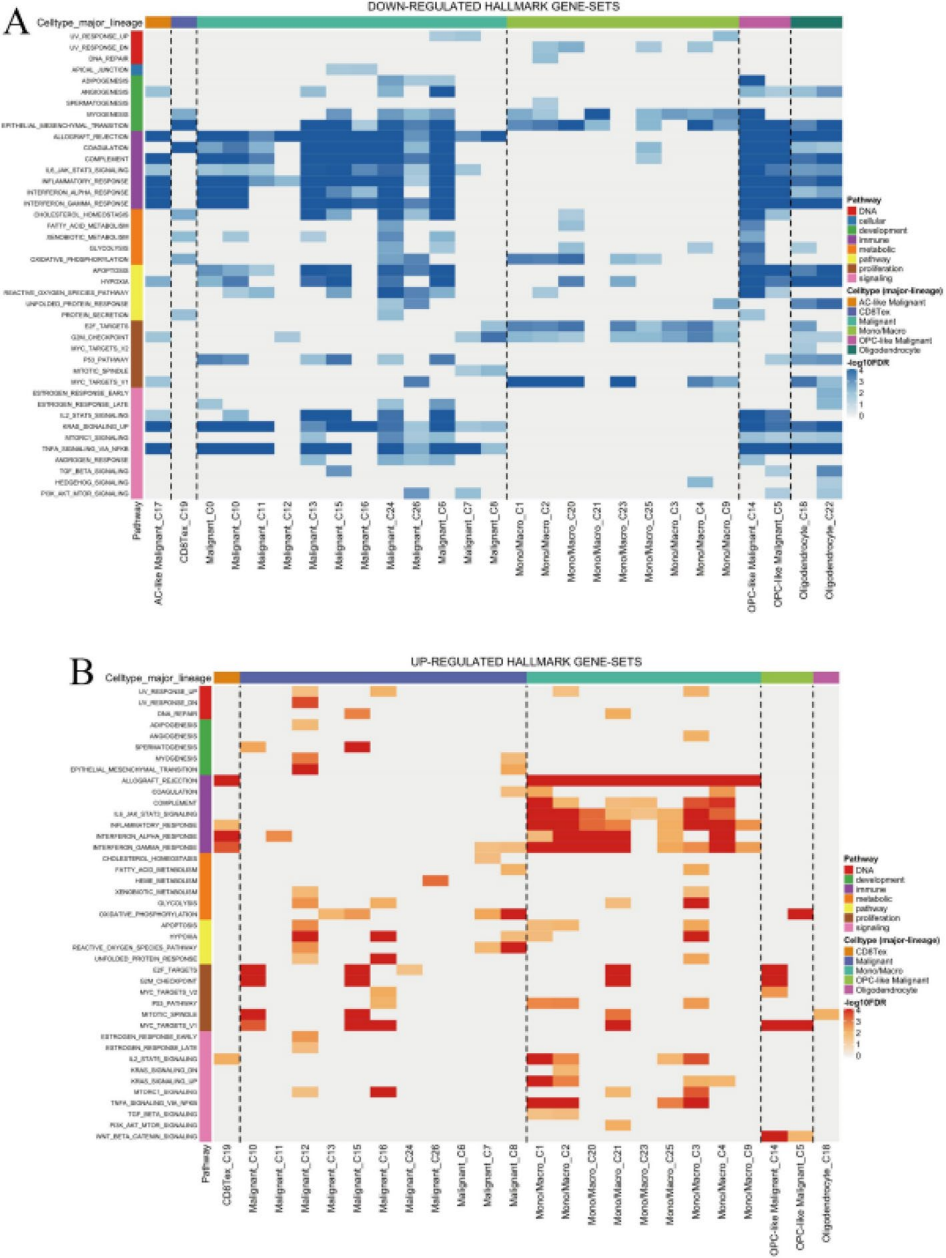
Hallmark gene sets that are differentially expressed across various cell lineages in glioma.** A** Down-regulated hallmark gene sets across various cell types, indicating decreased activity in specific pathways.** B** Up-regulated hallmark gene sets, highlighting increased activity in certain pathways across different cell types

Figure 10A illustrates pathway suppression patterns across cellular lineages, with blue intensity gradients reflecting down-regulation magnitude. Cell-type-specific pathway inactivation is evident in immune response and metabolic processes, indicating diminished functional activity in distinct cellular populations. Figure 10B depicts pathway activation profiles using red intensity scaling to represent up-regulation levels. Enhanced expression of proliferation and inflammatory gene sets demonstrates active biological engagement, with certain lineages showing pronounced pathway enrichment indicative of heightened cellular activity.

### The enrichment of hallmark pathways in different risk groups and their expression patterns in glioma

**Fig. 11:**
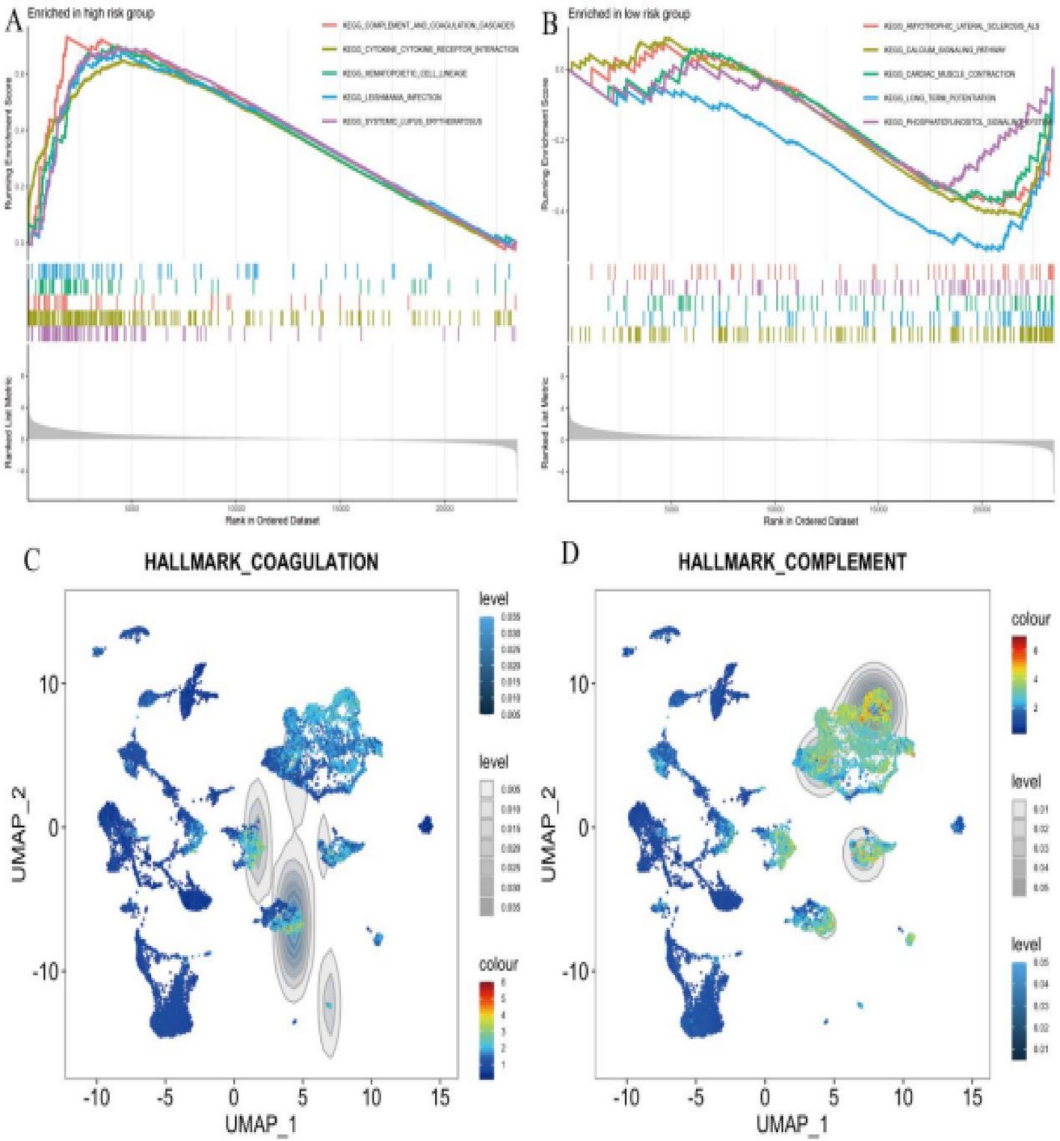
The enrichment of hallmark pathways in different risk groups and their expression patterns in glioma.** A** High-risk groups are enriched with specific pathways, such as DNA repair and inflammatory response.** B** Low-risk groups show enrichment in pathways like metabolism and immune regulation.** C**,** D** UMAP plots display the distribution of hallmark pathways, with coagulation and complement pathways showing distinct expression patterns across clusters

Figure 11A demonstrates pathway enrichment analysis for high-risk patients, revealing significant activation of inflammatory response and coagulation cascades. Enrichment score peaks identify maximum pathway activity within this patient subgroup. Figure 11B presents pathway enrichment profiles for low-risk patients, highlighting metabolic processes and immune regulation pathways. The enrichment distribution curves reflect distinct molecular signatures characterizing this favorable prognostic group. Figure 11C maps coagulation-related gene expression across cellular populations using density and color intensity visualization. Cluster-specific expression patterns indicate selective coagulation pathway activation within distinct tumor cell subsets. Figure 11D illustrates complement pathway gene expression distribution, with color intensity revealing cluster-specific activation patterns. These findings demonstrate complement system engagement in particular cellular contexts within the tumor microenvironment.

## Discussion

Spinal cord injury and gliomas represent two significant neurological challenges that may share underlying pathophysiological mechanisms. Spinal cord injuries can create an inflammatory microenvironment that, in some cases, may contribute to glial cell transformation. The trauma-induced disruption of normal tissue architecture and subsequent repair processes can potentially create conditions favorable for gliomagenesis in susceptible individuals. Gliomas, particularly when occurring in the spinal cord, can present with symptoms that mimic traumatic injury, including motor and sensory deficits, pain, and autonomic dysfunction. The relationship between prior spinal trauma and subsequent glioma development remains an area of active investigation, with some studies suggesting a possible association between chronic inflammation following injury and increased risk of neoplastic transformation in glial cells [17–21]. These findings highlights the necessity of better understanding glioma biology to discover better therapeutic alternatives.

Primary brain malignancies are predominantly represented by gliomas, with glioblastoma multiforme constituting the most lethal and therapeutically challenging variant. The clinical management of these neoplasms remains formidable due to their aggressive proliferative characteristics, extensive tissue infiltration, and pronounced molecular heterogeneity. Contemporary therapeutic modalities encompassing surgical resection, radiotherapy, and systemic chemotherapy have yielded only modest improvements in patient outcomes, with glioblastoma survival metrics frequently remaining below 15 months. This therapeutic plateau necessitates comprehensive investigation into the fundamental mechanisms governing glioma pathobiology to identify novel intervention strategies. Central to cancer pathophysiology, the tricarboxylic acid cycle represents a critical metabolic nexus orchestrating both bioenergetic production and anabolic precursor synthesis. Neoplastic transformation, particularly in glioma development, is characterized by profound metabolic reorganization that facilitates sustained proliferation and cellular survival under adverse conditions. Glioma cells demonstrate systematic modifications of TCA cycle functionality to accommodate their heightened metabolic demands and biosynthetic requirements. The cycle’s intermediate metabolites function as oncogenic mediators through diverse mechanisms, including direct modulation of cellular signaling cascades and epigenetic regulatory processes. Notably, alpha-ketoglutarate serves as an essential cofactor for dioxygenase enzymes responsible for chromatin modifications, including histone and DNA demethylation reactions that influence transcriptional programming. Clinical outcome associations with TCA cycle components reveal distinct prognostic patterns among key metabolic enzymes. GART, DHFR, and SHMT2 demonstrate hazard ratios exceeding unity, suggesting their elevated expression correlates with adverse clinical outcomes and enhanced disease progression. Conversely, IDH1 and OGDH exhibit hazard ratios below one, indicating potential protective roles or associations with favorable prognosis in specific patient subsets. The profound metabolic restructuring observed in gliomas manifests through characteristic enzymatic perturbations within the TCA cycle machinery. Mutations affecting isocitrate dehydrogenase enzymes exemplify this phenomenon, resulting in aberrant production of the oncometabolite D-2-hydroxyglutarate. This pathological metabolite functions as a competitive antagonist of alpha-ketoglutarate-dependent dioxygenases, consequently disrupting normal epigenetic regulation and promoting malignant transformation. These metabolic disturbances are intimately linked to the preservation of glioma stem cell populations and contribute significantly to the molecular heterogeneity that underlies therapeutic resistance mechanisms [22, 23].The advent of single-cell RNA sequencing (scRNA-seq) has opened new avenues for dissecting the glioma secretome and understanding the complex tumor microenvironment. This technology enables the isolation and examination of different cellular populations within tumors, shedding light on the heterogeneity and complexity of gliomas. scRNA-seq has identified various cell types in gliomas, including glioma stem cells (GSCs), differentiated tumor cells, and infiltrating immune cells. Understanding this therapeutic heterogeneity is essential for developing treatment modalities aimed at achieving remission.

The technology also provides insights into mechanisms of immune evasion and potential therapeutic targets by analyzing interactions between tumor cells and the immune microenvironment. Expression patterns of key metabolic genes (DHFR, GART, IDH1, OGDHL, SHMT2, SUCLG2) have been mapped across different cell populations, revealing distinct patterns: DHFR shows higher expression in specific clusters, suggesting functional importance in certain cell types or states; GART exhibits a highly localized expression pattern, implying selective cellular functions; IDH1 displays heterogeneous expression, likely related to its metabolic role in glioma; OGDHL shows localized high expression, potentially involved in energy metabolism; SHMT2’s variable expression may reflect its role in nucleotide biosynthesis; and SUCLG2 demonstrates high-expression clusters that suggest involvement in specific metabolic pathways. These insights help identify unique gene expression patterns associated with specific glioma subtypes and their metabolic states, offering potential biomarkers for prognosis and treatment response.

### Limitations

The most significant limitation of our study is the use of only four SNPs (rs1358980, rs217992, rs789990, and rs158541) as instrumental variables. While these SNPs met the genome-wide significance threshold (*p* < 5 × 10^−8) from the FinnGen R11 dataset, this number is substantially below the recommended threshold for robust mendelian randomization analysis.

## Conclusion

Combined metabolic profiling and single-cell sequencing provide a powerful new way to study glioma biology. Abstract: Background: The metabolic reprogramming effect of the TCA cycle and its dynamic influence on immune microenvironment has elucidated critical insights into tumor development and novel drug targets.

## Data Availability

The datasets generated and analyzed during the current study are available from the corresponding author upon reasonable request.
